# Arranging blood for elective thyroid surgeries: dilemma continues in the developing world

**DOI:** 10.1186/s13104-016-2315-9

**Published:** 2017-01-18

**Authors:** Salman Faridi, Aman Ahmad, Mirza Arshad Beg, Faisal Siddiqui, Muhammad Muzzammil Edhi, Mehmood Khan

**Affiliations:** 1 Department of General Surgery, Liaquat National Hospital and Medical College, Karachi, Pakistan; 2 Liaquat National Hospital and Medical College, Karachi, Pakistan

**Keywords:** Blood transfusion, Thyroidectomy

## Abstract

**Background:**

Cross matched blood is frequently ordered based on a subjective anticipation of blood loss for a procedure. Excessive blood arrangement and wastage overburdens the blood bank in terms of work load and storage of blood, increases cost of medical care and results in injudicious use of a limited resource. The aim of this short report is to assess the current practice for arranging cross matched blood in elective thyroid surgeries by comparing cross match to blood transfused ratio.

**Findings:**

Medical records for all patients from January 2009 to December 2014 undergoing thyroid surgery were retrieved and reviewed through electronic health information management system (HIMS). A total of 91 patients were included in the study, out of which 18 (19.7%) were male and 73 (80.2%) were female. A total of 107 units of blood were arranged and only 9 were transfused. 47 patients underwent a total thyroidectomy, while 44 underwent a hemithyroidectomy. The cross match to transfusion ratio came out to be 11.88.

**Conclusions:**

Routine arrangement of cross matched blood is not required in elective thyroid surgeries. All institutions should have a maximum blood ordering schedule planned for elective procedures, and blood products should be arranged accordingly to avoid unnecessary cross matching.

## Findings

### Introduction

Cross matched blood is frequently ordered based on a subjective anticipation of blood loss for a procedure. The maximum blood ordering schedule (MBOS) is a table of elective surgical procedures which lists the number of units of red cells routinely cross matched for them preoperatively [1]. The British society for hematology guidelines state that compatible blood should not generally be made available for surgery where the usage is <50% of units provided. This means a cross match to transfusion ratio (C:T) of 2:1 should be achieved [2].

In the absence of a maximum blood ordering schedule, there is a high chance of excess blood arrangement, which is not generally, required resulting in extra burden on the blood bank as well as the hospital resources in arranging for blood [2]. This practice not only adds to the already overburdened blood bank but also causes inconvenience to the patients and their families for arranging blood for an elective procedure. This has been proven in studies previously done as well as in local studies [3]. These findings highlight the need for a maximum blood ordering schedule.

The aim of this short report is to assess the current practice for arranging cross matched blood in elective thyroid surgeries by comparing cross match to blood transfused ratio (CT ratio) and to highlight the need of maximum blood ordering schedule in the developing world.

### Patient and methods

The study was approved by the institutions ethical review committee and written informed consents were obtained from all subjects for the surgery as well as for the purpose of consenting this study. 91 patients were included retrospectively in the study from January 2009 to December 2014.

Included were patients between 25 and 60 years of age undergone thyroid surgeries. Exclusion criteria were patients with anemia requiring preoperative transfusion and undergoing simultaneous additional surgical procedures. SPSS version 19.0 was used for descriptive statistics for concerned parameter.

### Results

A total of 91 patients were included in the study, out of which 18 (19.7%) were male and 73 (80.2%) were female. A total of 107 units of blood were arranged and only 9 units were transfused. The units were obtained from allogenic related donors as well as from allogenic unrelated donors to ensure continuous supply in the hospital. Of the 91 patients who were included in the study 6 patients were transfused postoperatively and of the 6 patients who were transfused postoperatively, 5 patients received 1 PRBC each, while 1 patient received 4 PRBC. 47 patients underwent a total thyroidectomy, while 44 underwent a hemithyroidectomy. The responsible doctor gave the orders for transfusion after reviewing the patient. The C:T ratio is 11.88.

### Discussion

According to this limited research the practice of arranging blood for transfusion results in depletion of the very valuable resource i.e. blood. The resources can be directed towards better management and provision for patients who require blood in emergency procedures [4]. In our setup, where there is a cost attached to ordering and arranging blood it is prudent for blood bank staff to utilize resources for the betterment of patients requiring blood in emergency. Our study further collaborates evidence in favor of similar studies in our part of the world, which shows that there is a need for implementation of maximum blood ordering schedule in our practice [5]. Policies regarding the use of maximum blood ordering schedule are not being adopted at our institution and this study will provide an impetus to further research on the topic [6].

Being a retrospective study, it warrants that more study on the subject is done and additional factors such as cost and total resources utilized are studied so as to highlight the importance of this very important issue [7]. Other elective cases may be studied and implementation of MBOS protocols is evaluated [8, 9]. A policy of group and hold in this regard is adhered to and the overall time for availability be communicated to the surgical team involved. This might help to allay fears and anxiety associated with non provision of blood in case of need.

### Conclusions

Our results indicate that routine arrangement of cross matched blood is not required in elective thyroid surgeries. All institutions should have their own institution-specific maximum blood ordering schedule for elective procedures, and blood products should be arranged accordingly to avoid unnecessary cross matching.

## Abbreviations

MBOS: maximum blood ordering schedule
CT ratio: cross match to blood transfused ratio
